# Antibiotic susceptibility testing and molecular characterization based on whole-genome sequencing of *Streptococcus pneumoniae* isolates from pediatric infections at the National Regional Medical Center of Southwest China during the COVID-19 pandemic

**DOI:** 10.3389/fpubh.2024.1490401

**Published:** 2024-12-10

**Authors:** Ziyi Yan, Chenglin Miao, Li Liu, Yunhan Fu, Xingxin Liu, Hong Li, Linghan Kuang, Yali Cui, Yongmei Jiang

**Affiliations:** ^1^Department of Laboratory Medicine, West China Second University Hospital, Sichuan University, Chengdu, China; ^2^Key Laboratory of Birth Defects and Related Diseases of Women and Children of Ministry of Education, West China Second University Hospital, Sichuan University, Chengdu, China; ^3^Department of Laboratory Medicine, Chengdu Hi-Tech Zone Hospital for Women and Children (Chengdu Hi-Tech Zone Hospital for Maternal and Child Healthcare), Chengdu, China; ^4^Department of Laboratory Medicine, Meishan Women and Children’s Hospital, Alliance Hospital of West China Second University Hospital, Sichuan University, Meishan, China; ^5^Department of Laboratory Medicine, West China Second University Hospital (Tianfu), Sichuan University/Sichuan Provincial Children’s Hospital, Meishan, China

**Keywords:** *Streptococcus pneumoniae*, antibiotic susceptibility, NGS, pediatrics, China, serotype, mlst, virulence

## Abstract

**Background:**

*Streptococcus pneumoniae* is a transmitted respiratory pathogen that causes high morbidity and mortality in children, especially those under 5 years of age. During the implementation of population control measures for COVID-19 in mainland China, the *Streptococcus pneumoniae* detection rate in pediatric patients decreased. However, with the second wave of the COVID-19 pandemic (2022), the incidence of pneumococcal disease (PD) and even invasive pneumococcal disease (IPD) began to rise again.

**Methods:**

This study was conducted from August 2022 to September 2023 at a national regional medical center based mainly in West China Second University Hospital, Sichuan University. The demographic and clinical characteristics of *S. pneumoniae*-infected pediatric patients were analyzed. All *S. pneumoniae* isolates were subjected to standardized clinical sample inoculation, culture, subculture, and identification procedures. Next-generation sequencing and analysis were used to determine serotypes and sequence types (STs) and evaluate antibiotic resistance- and virulence-related genes. Antimicrobial susceptibility was determined in AST dishes via the broth microdilution method.

**Results:**

The prevalent serotypes in the IPD patients were 14, 6A, and 23F, and the prevalent serotypes in the NIPD patients were 19F and 6A. A significant difference in the proportion of patients with serotype 14 was noted between the two groups. A total of 23 STs were identified and classified into 13 different GPSC lineages, including 4 novel STs (ST18449, ST18451, ST18464 and ST18466) and 1 novel allele (*ddl*1209). According to the interpretation breakpoints for non-meningitis infections, the resistance/nonsusceptibility rates of invasive isolates were as follows: penicillin (0.0%/8.3%), amoxicillin (0.0%/0.0%), cefotaxime (8.3%/16.6%), ceftriaxone (8.3%/8.3%), and cefepime (0.0%/8.3%). The resistance/nonsusceptibility rates of invasive isolates according to the meningitis breakpoints were as follows: penicillin (100.0%), cefotaxime (16.7%/33.4%), ceftriaxone (8.3%/50.0%), and cefepime (8.3%/66.7%). All the isolates were susceptible to rifampicin, levofloxacin, moxifloxacin, linezolid and vancomycin. In addition, the characteristics of the antibiotic resistance-related genes and virulence genes of serotype 19F were significantly different from those of the other serotypes.

**Conclusion:**

These data provide valuable information for understanding pediatric pneumococcal disease during the second outbreak of COVID-19 in Southwest China and will contribute to the prevention and treatment of *S. pneumoniae* infection.

## 1 Introduction

*Streptococcus pneumoniae* is a common extracellular pathogen worldwide. It often colonizes the mucosal surface of the upper respiratory tract of healthy people and is transmitted to susceptible populations through droplets, causing pneumococcal disease (PD) (1). After reaching the nasopharynx via droplets, *S. pneumoniae* may remain there, causing asymptomatic colonization, or may further invade the deep respiratory tract, causing pneumococcal pneumonia. Moreover, *S. pneumoniae* can release a variety of virulence factors, invade deep into tissue, break through the vascular endothelium, and cause pneumococcal sepsis. Furthermore, *S. pneumoniae* can continue to accumulate in the peripheral blood and multiply in the brain, causing pneumococcal meningitis. In clinical practice, *S. pneumoniae* from the nasopharynx has also been found to directly invade the brain after head trauma (2). Currently, *S. pneumoniae* is not only the leading cause of bacterial pneumonia in children but also the leading cause of sepsis and meningitis in children globally; this type of infection is referred to as invasive pneumococcal disease (IPD) (3).

At the end of 2019, COVID-19 was first discovered in China, and the first wave of the global pandemic occurred. From 2019 to 2022, the Chinese government implemented a series of strict population control measures to curb the spread of COVID-19, with each area adopting “zero infection” as the primary medical goal. The measures taken to cut off the transmission routes of respiratory pathogens also reduced the incidence of bacterial respiratory infections, such as pneumococcal disease, in children. However, since 2022, a second wave of the COVID-19 pandemic has occurred in various areas of mainland China despite the “early diagnosis and early isolation” strategy for COVID-19, which has lasted for more than 2 years. As population control measures been downgraded and even lifted, the incidence of transmitted respiratory bacterial infections in children, including *S. pneumoniae* infections, has rebounded rapidly.

Widespread vaccination with pneumococcal conjugate vaccines (PCVs) can not only reduce the infection rates of the serotypes they cover in the population but also protect against serotypes with high resistance frequencies (4). As one of the few vaccines designed to target resistant pathogens directly, the widespread use of PCVs has led to decreases in the proportions of *S. pneumoniae* exhibiting nonsusceptibility to penicillin, sulfamethoxazole-trimethoprim and third-generation cephalosporins on average across all regions (5). However, the increase in non-PCV13 serotypes associated with antibiotic resistance is concerning, especially during and after the COVID-19 pandemic (6).

As early as 2008, a pneumococcal polysaccharide conjugate vaccine (PCV) for children under 2 years of age was introduced in mainland China. The 7-valent pneumococcal polysaccharide conjugate vaccine (PCV-7) was subsequently delisted in mainland China in 2015, and the 13-valent pneumococcal polysaccharide conjugate vaccine (PCV-13) was launched in mainland China in 2017. However, since PCVs have not been included in the national immunization program in mainland China to date, the vaccination rates are low, as these are self-purchased vaccines that cost at least US$200 for the complete four-dose course. In 2021, the final-dose coverage of PCV-13 in mainland China was 12.47%, and in some provinces, it was only 1.17%, showing obvious interregional differences. In contrast, the final-dose coverage rates in the United States, Australia, the European Region and India were 92.0, 96.27, 82 and 69.3%, respectively (7). Therefore, Chinese children not only lack passive immunity against *S. pneumoniae* due to COVID-19 population control measures but also lack active immunity due to the low PCV-13 vaccination rate. During this period, changes in antibiotic susceptibility and the molecular characteristics of recirculating *S. pneumoniae* isolates are of concern.

In this study, we performed a cross-sectional study on PD in children during the second outbreak of COVID-19 in China. Whole-genome sequencing of *S. pneumoniae* isolates and antimicrobial susceptibility experiments were used to evaluate and analyze the distribution characteristics of their sensitivity to 16 antimicrobial agents, serotypes, sequence types (STs), antibiotic resistance-related genes, and virulence-related genes in Southwest China, aiming to describe the prevalence of *S. pneumoniae* in pediatric patients in this area.

## 2 Materials and methods

### 2.1 Patients

This study was conducted from August 2022 to September 2023 (during the second outbreak of COVID-19 in China) at West China Second University Hospital/National Regional Medical Center (Southwest China), which is one of the largest specialized hospitals for women and children in China. The hospital’s clinical laboratory has been accredited by the College of American Pathologists (CAP) and the ISO15189 accreditation standard.

The enrolled patients were divided into two groups according to the following eligibility criteria: (1) pediatric patients (≤12 years) diagnosed with pneumococcal meningitis and/or sepsis according to the International Classification of Diseases 10th Revision (ICD-10), with *S. pneumoniae* isolated from their peripheral blood and/or cerebrospinal fluid (CSF), as the invasive pneumococcal disease (IPD) group; and (2) pediatric patients (≤12 years) diagnosed with pneumococcal pneumonia according to the ICD-10, with *S. pneumoniae* isolated from their sputum, as the noninvasive pneumococcal disease (NIPD) group (control group). Stratified random sampling was performed on the basis of the date of consultation (within 30 days) and patient age (within 1 year) among the NIPD patients, and the NIPD patients were matched with the IPD patients at a ratio of 1:2. All patients had not previously received pneumococcal vaccination.

### 2.2 Isolate culture and identification

Isolates of *S. pneumoniae* were collected, cultured and identified in accordance with the requirements for clinical procedures as previously reported (8). In brief, specimens were collected by specialized sample collection personnel or physicians, following ISO/TS 20658:2017 Medical Laboratories: Requirements for collection, transport, receipt, and handling of samples (blood, sputum, cerebrospinal fluid, chest drainage and secretions). Peripheral blood and cerebrospinal fluid were cultured in vials with the BD BACTEC™ FX system (BD Medical Technology, NJ, USA) and then subcultured on Columbia agar +5% sheep blood plates (Autobio, Zhengzhou, China). Sputum was isolated on Columbia agar +5% sheep blood plates (Autobio, Zhengzhou, China) and incubated at 35°C for 24–48 h in a 5% carbon dioxide (CO_2_) environment. All the isolates were identified via matrix-assisted laser desorption ionization time-of-flight mass spectrometry (MALDI-TOF MS; Vitek MS system; BioMerieux, Rhône, France), and the isolates were confirmed via optochin resistance, bile solubility and PubMLST whole-genome signature sequence alignment.

### 2.3 Genome sequencing, assembly and annotation

Genome sequencing of *S. pneumoniae* isolates was performed as previously reported (9). In brief, genomic DNA was extracted via a QIAamp DNA Minikit (Qiagen, Hilden, Germany) with sequencing libraries generated via the NEBNext® Ultra™ DNA Library Prep Kit for Illumina (New England Biolabs, NEB, USA) and then sequenced via the Illumina NovaSeq PE150 platform (Illumina, San Diego, CA, USA) with approximately 200× coverage (read length 150 bp, paired-end).

The genome data were assembled and annotated as previously reported (10). In brief, the genome data were assembled with SPAdes software (v3.14.1) (11) and then annotated with Prokka software (v1.14.5) (12). All of the assembled genomes were submitted to GenBank and approved (PRJNA1045333).

### 2.4 Molecular serotyping, sequence typing and GPSC lineages

The molecular serotypes of the isolates were identified via pneumococcal capsule typing (PneumoCaT) software (v1.2.1) (13) with reference to the capsular locus sequences and the capsular type variant (CTV) database. The sequence types (STs) of the isolates were identified via mlst software (v2.19.0) based on comparison with the multilocus sequence typing (MLST) database (14). Novel ST alleles and profiles were submitted to the pneumococcal MLST database to assign new numbers. The global pneumococcal sequence cluster (GPSC) and clonal complexes (CCs) were identified via PathogenWatch^1^ on the basis of the international genomic definition of pneumococcal lineages (15, 16).

### 2.5 Phylogenetic analysis

The phylogenetic analysis of *S. pneumoniae* was conducted as described in our previous study (10). In brief, the annotated genomes were analyzed via Roary software (v3.13.0) to create a multiFASTA alignment of core genes (>99%) (17). After deleting duplicate sites via snp-sites software (18), maximum-likelihood trees were constructed via RAxML software (v8.2.10) using the GTRGAMMA method (19). The phylogenetic tree was subsequently visualized and annotated by Interactive Tree of Life (iTOL, v6.9.1) (20).

### 2.6 Antibiotic resistance-related gene and virulence gene analysis

Antibiotic resistance-related genes were analyzed via ABRicate software (v1.0.1) using NCBI AMRFinderPlus (21), the Comprehensive Antibiotic Resistance Database (CARD) (22), and Antibiotic Resistance Gene-ANNOTation (ARG-ANNOT) (23). With the assistance of Pathogenwatch (See footnote 1), amr-libraries (PAARSNP AMR-Library 1313 version 0.0.16) were used to analyze trimethoprim-sulfamethoxazole resistance determinants and *tetM* allele types, and Spn_Scripts_Reference^3^ was used for PBP types. Furthermore, PBP transpeptidase signatures were used to predict β-lactam resistance levels (24). Virulence genes were analyzed via ABRicate software (v1.0.1) using the virulence factor database (VFDB) (25), which included genes from the following groups: exotoxin, exoenzyme, immune modulation, nutritional/metabolic factors, adherence, pilus-1 and pilus-2.

### 2.7 Antibiotic susceptibility tests

Antibiotic susceptibility tests (ASTs) were performed via the broth turbidimetry method using AST dishes (TDR STR-AST, Mindray, China) as described in our previous report (9). The antimicrobial agents used included penicillin (PEN), amoxicillin (AMX), cefuroxime (CXM), cefotaxime (CTX), ceftriaxone (CRO), cefepime (FEP), meropenem (MEM), rifampin (RIF), levofloxacin (LVX), moxifloxacin (MXF), trimethoprim/sulfamethoxazole (SXT), tetracycline (TCY), clindamycin (CLI), erythromycin (ERY), linezolid (LNZ) and vancomycin (VAN). Quality control analysis was performed using *S. pneumoniae* ATCC 49619. The operational processes and interpretation of the results were performed according to the manufacturer’s instructions and the Clinical and Laboratory Standards Institute (CLSI) M100: Performance Standards for Antimicrobial Susceptibility Testing, 34th Edition. For PEN, AMX, CXM, CTX, CRO and FEP, the interpretation of antibiotic susceptibility was dependent on their administration routes. For patients with meningitis, non-meningitis breakpoints are not reported clinically. Moreover, the results of this study were used only for comparisons among isolates and did not involve clinical interpretation.

### 2.8 Statistical analysis

Statistical Package for Social Science (SPSS) software for Windows was used to assess the statistical significance of the data (version 22.0; Chicago, IL, USA), as previously reported (10). In brief, the chi-square test and Fisher’s exact test were used to evaluate qualitative data and the composition ratio, and the Z test (using the Bonferroni method to correct *p* values) was used to evaluate whether the difference among subgroups was significant. The Mann–Whitney U test and Kolmogorov–Smirnov test were used to conduct nonparametric analyses. T tests and analysis of variance (ANOVA) were used to evaluate quantitative data, and the Bonferroni method was used to evaluate whether the difference among subgroups was significant. p values <0.05 were considered indicative of statistical significance.

## 3 Results

### 3.1 Demographic and clinical characteristics

A total of 12 cases of invasive *S. pneumoniae* infection were identified from 2022 to 2023 during the second outbreak of COVID-19 in China, and all of the cases were sepsis secondary to pneumococcal pneumonia, including 4 meningitis cases. Among them, 9 patients were male (75.0%), with a median age (P25-P75) of 1.92 (0.92–3.00) years. The samples included CSF and peripheral blood, and all of the patients had a favorable prognosis (Table 1).

**Table 1:** Demographic and clinical characteristics of 36 patients with pneumococcal diseases.

Characteristics	IPD		NIPD		***p***
	No. of patients	(%)	No. of patients	(%)	
Sex					
Male	9	75.0	14	58.3	0.468
Female	3	25.0	10	41.7	
Age (years)					
≤3	10	83.3	19	79.2	
4–5	1	8.3	5	20.8	
6–12	1	8.3	0	0.0	
Median age (P25-P75)	1.92 (0.92–3.00)		1.92 (1.25–3.00)		0.973
Sample type					
Cerebrospinal fluid (CSF)	4	33.3	0	0.0	
Blood	8	66.7	0	0.0	
Sputum	0	0.0	24	100.0	
Diagnosis					
Sepsis & pneumonia	12	100.0	0	0.0	
with meningitis	4	33.3	0	0.0	
with endocarditis	1	8.3	0	0.0	
Pneumonia			24	100.0	
co-SARS-CoV-2 infection	0	0.0	3	12.5	
co-Influenza A virus infection	0	0.0	1	4.2	
co-Parainfluenza virus infection	0	0.0	1	4.2	
co-*Mycoplasma pneumoniae* infection	0	0.0	3	12.5	
Elevated CRP levels in blood					
Yes	11	91.7	9	37.5	**0.004**
No	1	8.3	15	62.5	
Elevated WBC levels in blood					
Yes	5	41.7	3	12.5	**0.047**
No	7	58.3	21	87.5	
Elevated WBC levels in CSF (meningitis)					
Yes	4	100.0			
No	0	0.0			
Prognosis					
Cured	7	58.3	18	75.0	
Improved	5	41.7	6	25.0	
Exacerbation or death	0	0.0	0	0.0	
Total	12	100.0	24	100.0	

Among the NIPD patients, stratified random sampling was performed on the basis of the date of consultation (within 30 days) and the patient’s age (within 1 year), and these patients were matched with the IPD patients at a ratio of 1:2. No significant differences in demographic characteristics were noted between the two groups. However, the proportion of patients with elevated C-reactive protein (CRP) levels in the IPD group was significantly greater than that in the NIPD group. Because the reference intervals of peripheral leukocytes in children of different ages vary greatly, only whether the value is elevated is recorded. Bold: This *p* value indicates that the data are significantly different.

Twenty-four patients with NIPD were matched with these 12 patients with IPD at a ratio of 2:1 via stratified random sampling; the median age (P25-P75) was 1.92 (1.25–3.00) years. All of the NIPD patients had pneumonia, with *S. pneumoniae* isolated from their sputum sample, including 5 patients with co-viral infection and 3 patients with co-*Mycoplasma pneumoniae* infection. All of these patients had a favorable prognosis (Table 1). No significant differences in sex or age composition were noted between the two groups according to Fisher’s exact test or the Mann–Whitney U test. Almost all the IPD patients had elevated C-reactive protein (CRP) levels during their course of disease, and these levels were significantly greater than those in the NIPD patients (91.7% vs. 37.5%, *p* = 0.004). All patients with meningitis had elevated WBC counts in the CSF. Although the incidence of elevated peripheral leukocyte counts in IPD patients was significantly greater than that in NIPD patients (41.7% vs. 12.5%, *p* = 0.047), the diagnostic sensitivity was low because the reference intervals of peripheral leukocyte count in children of different age ranges vary greatly.

### 3.2 Distribution of serotypes, STs and GPSC lineages

**Figure 1:**
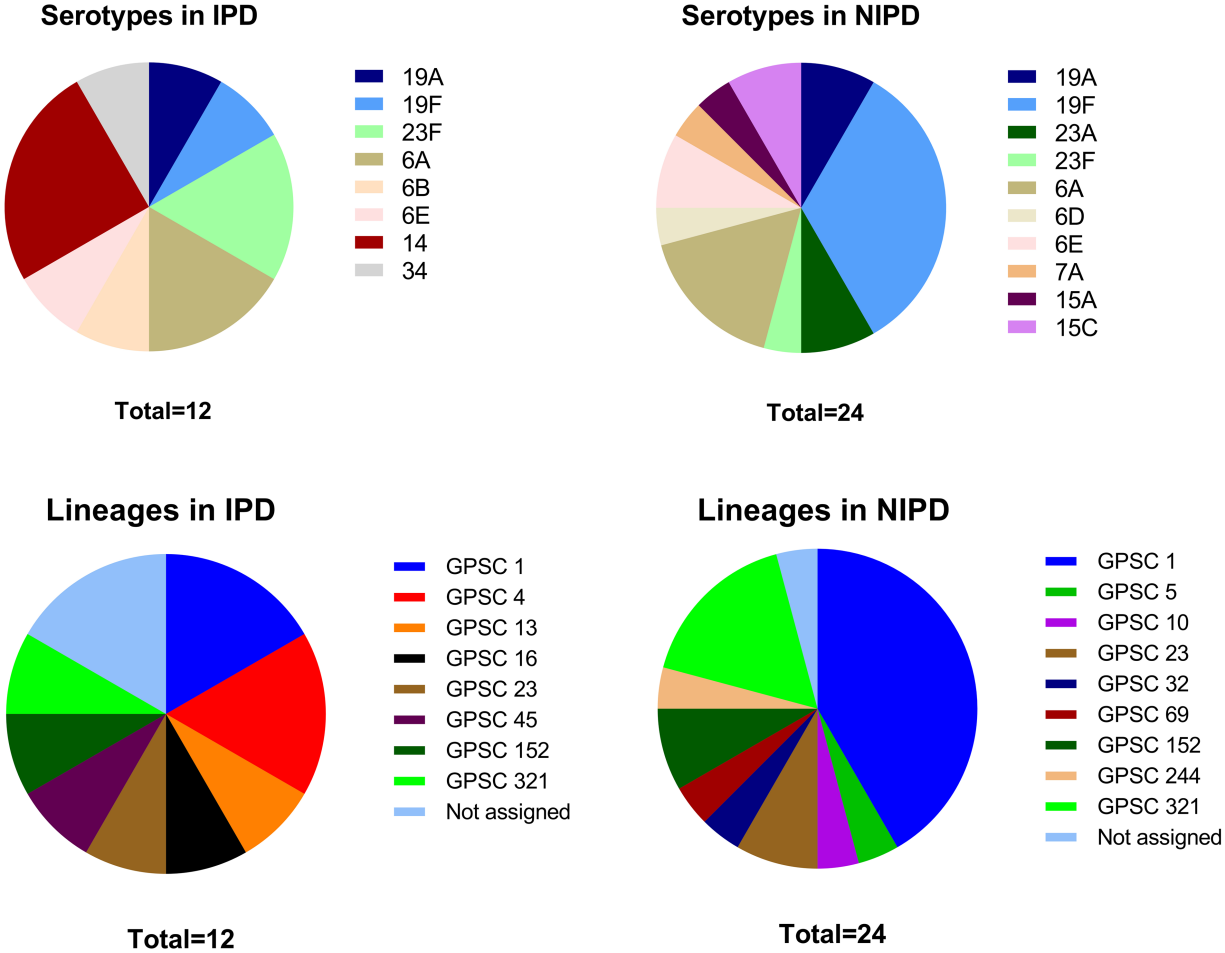
Serotype and GPSC lineage distributions in IPD patients and NIPD patients. *: The proportion of this serotype significantly differed between the two groups (*p* < 0.050).

A total of 13 different serotypes were detected in this study (6A, 6B, 6D, 6E, 7A, 14, 15A, 15C, 19A, 19F, 23A, 23F and 34), including 8 serotypes in IPD patients and 10 serotypes in NIPD patients (Figure 1). Compared with NIPD cases, in which serotype 19F was the dominant serotype (33.3%), the serotype distribution in IPD cases was more balanced, with serotype 14 accounting for 25.0% and ranking first. In addition to serotype 14, serotypes 19A (8.3%), 19F (8.3%), 23F (16.7%), 6A (16.7%), 6B (8.3%), 6E (8.3%) and 34 (8.3%) were also isolated from IPD patients. Fisher’s exact test revealed that the proportion of serotype 14 cases among IPD cases was significantly greater than that among NIPD cases (25.0% vs. 0.0%, *p* = 0.031). Although the proportion of serotypes within PCV-13 was greater in IPD cases than in NIPD cases, this difference was not significant (91.7% vs. 70.8%, *p* = 0.224).

**Figure 2:**
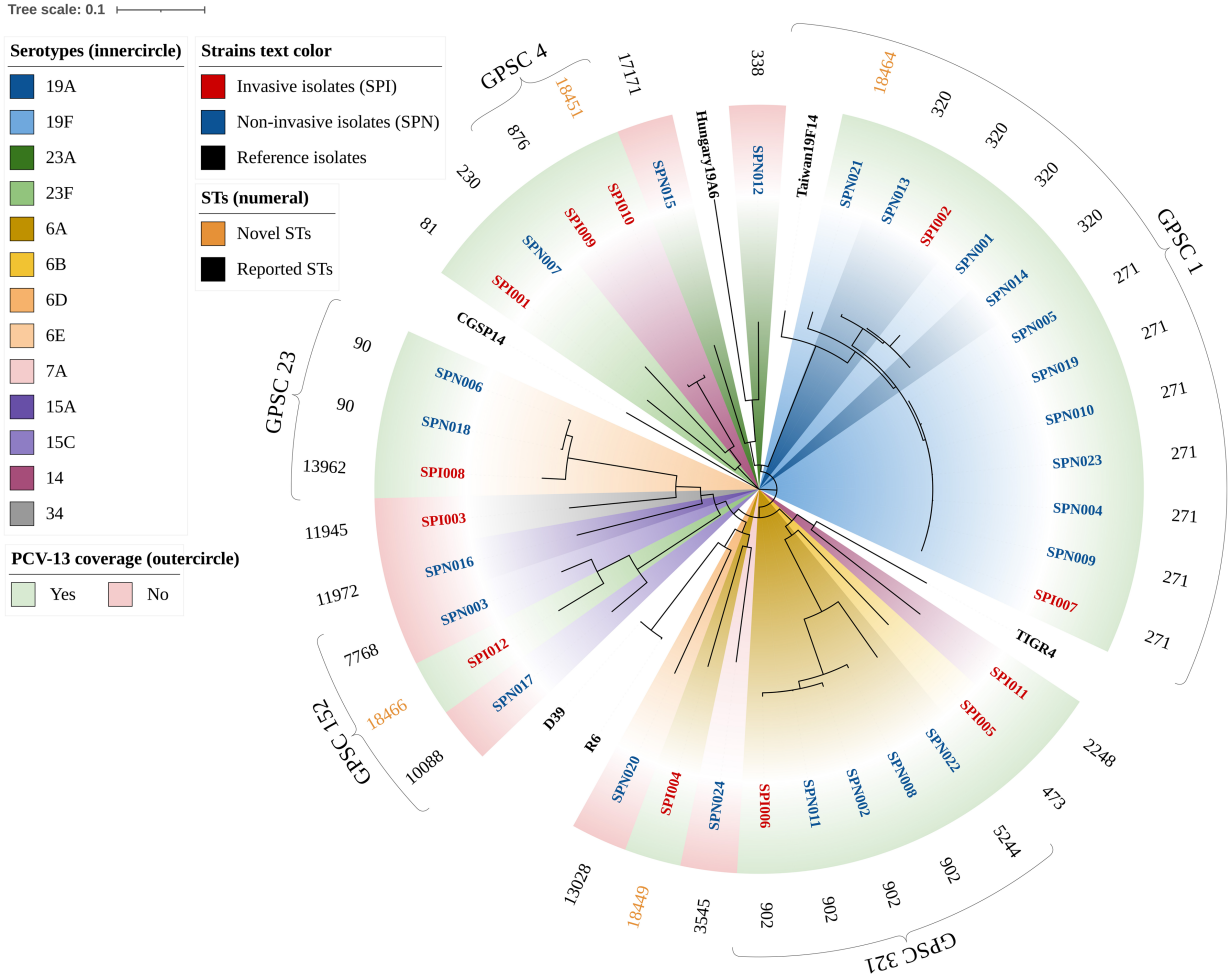
Phylogenetic tree of 36 clinical isolates of *S. pneumoniae* (circular). The branches are labeled with the isolate name, and the color of the label indicates the strain type (e.g., red text represents invasive isolates). The standard strains of *S. pneumoniae*, including R6, D39, TIGR4, CGSP14, Hungary^19A^-6, and Taiwan^19F^-14, were used as reference genomes. The inner conical color block is annotated with the serotype of each isolate (e.g., dark blue represents serotype 19A). The outer rectangular color block indicates whether the serotype of the isolate is covered by the PCV-13 vaccine. The outer text is annotated with the STs of the isolate, and novel STs are reported in gold.

A total of 23 STs were identified via MLST, including 4 novel STs (ST18449, ST18451, ST18464, and ST18466), with 1 novel allele (*ddl*1209) and 3 novel profiles^4^ (Figure 2). GPSC analysis classified these strains into 13 different GPSC lineages (1, 4, 5, 10, 13, 16, 23, 32, 45, 69, 152, 244, and 321), among which GPSC 1 (CC320) was dominant and was concentrated mainly in NIPD patient isolates (Figure 1). Similar to the distribution of serotypes, the distribution of STs in IPD cases was more dispersed, with the major lineages GPSC 1 (CC320) and GPSC 4 (CC199) accounting for 16.7% each, whereas 41.7% of the isolates in NIPD cases belonged to GPSC 1 (CC320). Phylogenetic analysis revealed that GPSC 1 (CC320) and GPSC 321 (CC902) were the major clades, with serotypes 19A/19F and 6A/6B, respectively. Among the novel STs, ST18464, ST18451, ST18466, and ST18449 belonged to GPSC 1 (CC320), GPSC 4 (CC199), GPSC 152 and singletons (not assigned), respectively, with serotypes 19F, 14, 23F, and 6A, respectively (Figure 2).

### 3.3 Antibiotic susceptibility phenotype

**Figure 3:**
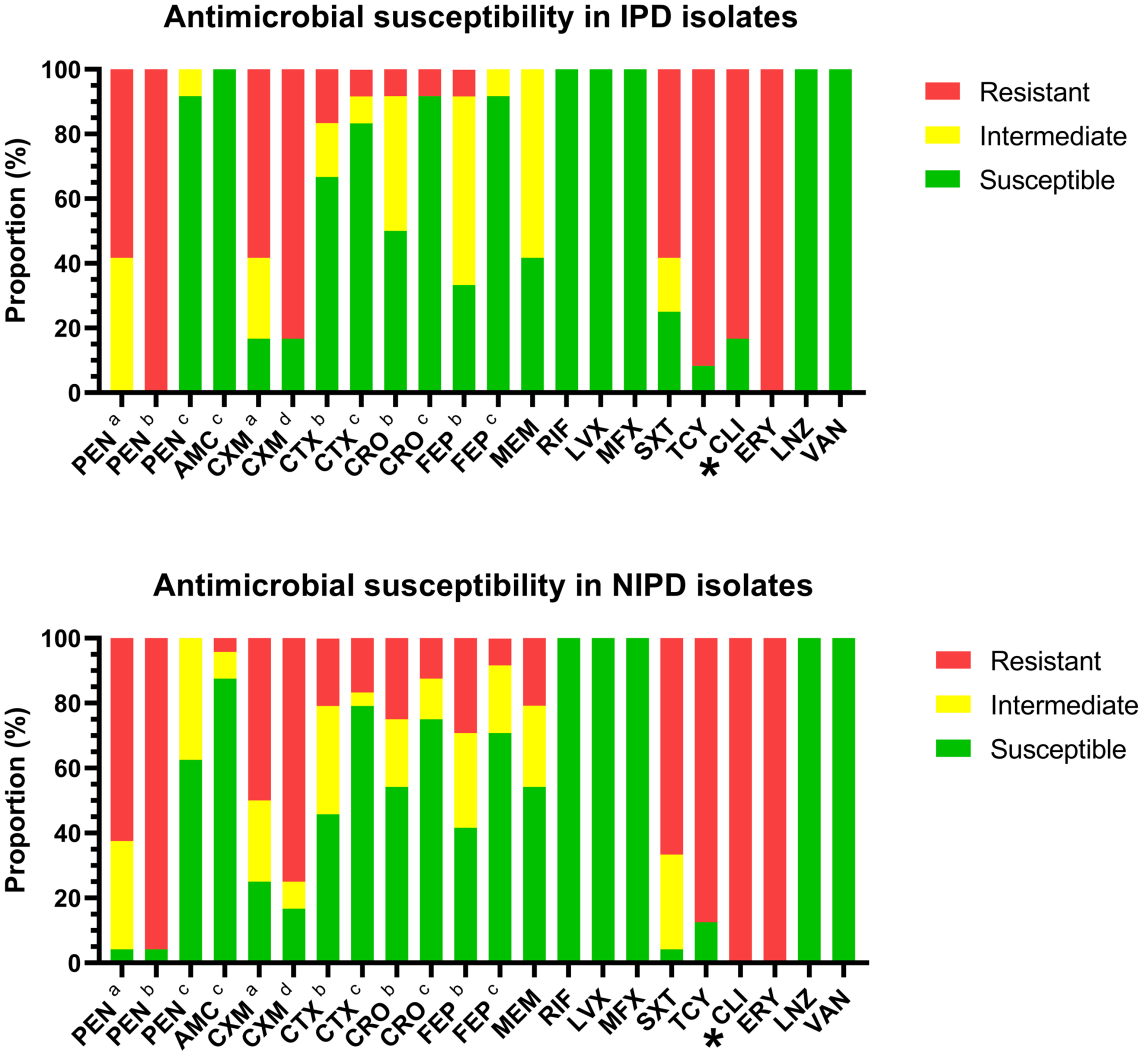
Antibiotic susceptibility of IPD and NIPD isolates. The results were interpreted according to the CLSI criteria on the basis of the minimum inhibitory concentration of each antimicrobial agent. (a) Oral breakpoint. (b) Meningitis breakpoint. (c) Non-meningitis breakpoint. (d) Intravenous breakpoint. *: The resistance interpretation results for this antimicrobial agent in IPD isolates and NIPD isolates were significantly different (*p* < 0.050).

According to the CLSI interpretation criteria for antibiotic susceptibility tests, all of these isolates were susceptible to rifampin, levofloxacin, moxifloxacin, linezolid and vancomycin. The resistance proportions of the IPD isolates against each antimicrobial agent were as follows: (1) penicillin (oral/ meningitis/non-meningitis): 58.3%/100.0%/0.0%; (2) amoxicillin (non-meningitis): 0.0%; (3) cefuroxime (oral/intravenous): 58.3%/83.3%; (4) cefotaxime (meningitis/non-meningitis): 16.7%/8.3%; (5) ceftriaxone (meningitis/non-meningitis): 8.3%/8.3%; (6) cefepime (meningitis/non-meningitis): 8.3%/0.0%; (7) meropenem: 0.0%; (8) trimethoprim/sulfamethoxazole: 58.3%; (9) tetracycline: 91.7%; (10) clindamycin: 83.3%; and (11) erythromycin: 100.0% (Figure 3, upper portion). The resistance proportions of the NIPD isolates were as follows: (1) penicillin (oral/meningitis/non-meningitis): 62.5%/95.8%/0.0%; (2) amoxicillin (non-meningitis): 4.2%; (3) cefuroxime (oral/intravenous): 50.0%/75.0%; (4) cefotaxime (meningitis/non-meningitis): 20.8%/16.7%; (5) ceftriaxone (meningitis/non-meningitis): 25.0%/12.5%; (6) cefepime (meningitis/non-meningitis): 29.2%/8.3%; (7) meropenem: 20.8%; (8) trimethoprim/sulfamethoxazole: 66.6%; (9) tetracycline: 87.5%; (10) clindamycin: 100.0%; and (11) erythromycin: 100.0% (Figure 3, lower portion). According to the direct interpretation of various breakpoints, the resistance rate of IPD isolates to clindamycin was significantly lower than that of NIPD isolates (*p* = 0.040).

The distributions of the minimum inhibitory concentrations (MICs) of the antimicrobial agents are shown in Table 2. The concentrations of each antimicrobial agent that inhibited 90% of the IPD isolates (MIC_90_) were as follows: (1) penicillin: 2 μg/ml; (2) amoxicillin: ≤ 2/1 μg/ml; (3) cefuroxime: 4 μg/ml; (4) cefotaxime: ≤ 0.5 μg/ml; (5) ceftriaxone: ≤ 0.5 μg/ml; (6) cefepime: 1 μg/ml; (7) meropenem: 0.5 μg/ml; (8) rifampin: ≤ 0.5 μg/ml; (9) levofloxacin: ≤ 2 μg/ml; (10) moxifloxacin: ≤ 0.5 μg/ml; (11) trimethoprim/sulfamethoxazole: 4/76 μg/ml; (12) tetracycline: 16 μg/ml; (13) clindamycin: ≥ 8 μg/ml; (14) erythromycin: ≥ 16 μg/ml; (15) linezolid: ≤ 2 μg/ml; and (23) vancomycin: ≤ 0.5 μg/ml. The Mann–Whitney U test and Kolmogorov–Smirnov test revealed significant differences in the distributions of the MICs of clindamycin (P_[M-W]_ = 0.015, P_[K-S]_ = 0.034) and erythromycin (P_[M-W]_ = 0.016, P_[K-S]_ = 0.034) in the IPD and NIPD isolates.

**Table 2:** Minimum inhibitory concentration (MIC) distributions of 36 *Streptococcus pneumoniae* isolates in this study.

		Minimum Inhibitory Concentration (MIC, μg/ml)								Susceptibility			MIC_50_	MIC_90_	*P_(M-W)_*	*P_(K-S)_*
Antibiotics	Cases	≤0.03	0.12	0.25	0.5	1	2	4	≥8	S%	I%	R%	μg/ml	μg/ml		
PEN^a^	IPD	0	0	1	2	2	6	1	0	0.0%	41.7%	58.3%	2	2	0.331	0.235
	NIPD	1	0	1	4	3	6	9	0	4.2%	33.3%	62.5%	2	4		
PEN^b^	IPD	0	0	1	2	2	6	1	0	0.0%	NA	100.0%	2	2	0.331	0.235
	NIPD	1	0	1	4	3	6	9	0	4.2%	NA	95.8%	2	4		
PEN^c^	IPD	0	0	1	2	2	6	1	0	91.7%	8.3%	0.0%	2	2	0.331	0.235
	NIPD	1	0	1	4	3	6	9	0	62.5%	37.5%	0.0%	2	4		

Antibiotics	Cases	≤0.25	0.5	1	2	4	8	16	≥32	S%	I%	R%	μg/ml	μg/ml		
AMX^c^	IPD			-	12	0	0	0	0	100.0%	0.0%	0.0%	≤2/1	≤2/1	0.208	0.536
	NIPD			-	21	2	1	0	0	87.5%	8.3%	4.2%	≤2/1	4/2		
CXM^a^	IPD	-	2	0	3	7	0	-		16.7%	25.0%	58.3%	4	4	0.523	0.074
	NIPD	-	4	2	6	3	9	-		25.0%	25.0%	50.0%	2	≥8		
CXM^d^	IPD	-	2	0	3	7	0	-		16.7%	0.0%	83.3%	4	4	0.523	0.074
	NIPD	-	4	2	6	3	9	-		16.7%	8.3%	75.0%	2	≥8		
CTX^b^	IPD	-	8	2	1	1	0	-		66.7%	16.7%	16.7%	≤0.5	2	0.286	0.463
	NIPD	-	11	8	1	3	1	-		45.8%	33.3%	20.8%	1	4		
CTX^c^	IPD	-	8	2	1	1	0	-		83.3%	8.3%	8.3%	≤0.5	2	0.286	0.463
	NIPD	-	11	8	1	3	1	-		79.1%	4.2%	16.7%	1	4		
CRO^b^	IPD	-	6	5	0	1	0	-		50.0%	41.7%	8.3%	≤0.5	1	0.854	0.699
	NIPD	-	13	5	3	3	0	-		54.2%	20.8%	25.0%	≤0.5	4		
CRO^c^	IPD	-	6	5	0	1	0	-		91.7%	0.0%	8.3%	≤0.5	1	0.854	0.699
	NIPD	-	13	5	3	3	0	-		75.0%	12.5%	12.5%	≤0.5	4		
FEP^b^	IPD	-	4	7	1	0	0	-		33.3%	58.3%	8.3%	1	1	0.720	0.436
	NIPD	-	10	7	5	1	1	-		41.6%	29.2%	29.2%	1	2		
FEP^c^	IPD	-	4	7	1	0	0	-		91.7%	8.3%	0.0%	1	1	0.720	0.436
	NIPD	-	10	7	5	1	1	-		70.8%	20.8%	8.3%	1	2		
MEM	IPD	5	7	0	0	0	0	0	-	41.7%	58.3%	0.0%	0.5	0.5	0.985	0.377
	NIPD	13	6	3	2	0	0	0	-	54.2%	25.0%	20.8%	≤0.25	1		
RIF	IPD	-	12	0	0	0	-			100.0%	0.0%	0.0%	≤0.5	≤0.5	1.000	1.000
	NIPD	-	24	0	0	0	-			100.0%	0.0%	0.0%	≤0.5	≤0.5		
LVX	IPD			-	12	0	0	0	-	100.0%	0.0%	0.0%	≤2	≤2	1.000	1.000
	NIPD			-	24	0	0	0	-	100.0%	0.0%	0.0%	≤2	≤2		
MFX	IPD	-	12	0	0	0	-			100.0%	0.0%	0.0%	≤0.5	≤0.5	1.000	1.000
	NIPD	-	24	0	0	0	-			100.0%	0.0%	0.0%	≤0.5	≤0.5		
SXT^e^	IPD	-	3	1	1	1	6	-		25.0%	16.7%	58.3%	4/76	≥8/152	0.416	0.479
	NIPD	-	1	4	3	2	14	-		4.2%	29.2%	66.6%	≥8/152	≥8/152		
TCY	IPD	-	1	0	0	1	2	3	5	8.3%	0.0%	91.7%	16	≥32	0.413	0.643
	NIPD	-	3	0	0	0	1	7	13	12.5%	0.0%	87.5%	≥32	≥32		
CLI	IPD	3	0	0	0	1	8	-		16.7%	0.0%	83.3%	≥8	≥8	**0.015**	**0.034**
	NIPD	0	0	0	0	1	23	-		0.0%	0.0%	100.0%	≥8	≥8		
ERY	IPD	0	0	0	2	0	2	8	-	0.0%	0.0%	100.0%	≥16	≥16	**0.016**	**0.034**
	NIPD	0	0	0	0	0	1	23	-	0.0%	0.0%	100.0%	≥16	≥16		
LNZ	IPD			-	12	0	0	0	-	100.0%	NA	0.0%	≤2	≤2	1.000	1.000
	NIPD			-	24	0	0	0	-	100.0%	NA	0.0%	≤2	≤2		
VAN	IPD	-	12	0	0	0	0	0	0	100.0%	NA	0.0%	≤0.5	≤0.5	1.000	1.000
	NIPD	-	24	0	0	0	0	0	0	100.0%	NA	0.0%	≤0.5	≤0.5		

a. Oral administration route. b. Interpretation on the basis of breakpoints for the treatment of meningitis (i.v.). c. Interpretation on the basis of breakpoints for the treatment of non-meningitis infections (i.v.). d. Administration via intravenous infusion. e. According to the sulfamethoxazole concentration, the mass ratio of sulfamethoxazole to trimethoprim was 1:19. The dashed lines indicate the breakpoints from susceptible to susceptible with increased exposure levels of antibiotics. The solid lines indicate the breakpoints from susceptibility with increased exposure to resistant levels of antibiotics. “-” indicates the boundary of the antibiotic dilution concentration in the antibiotic susceptibility test. NA: Not applicable, indicating that this antibiotic has no susceptibility with increased exposure classification in the CLSI breakpoint interpretation. *P_(M-W)_*: The Mann–Whitney U test was used to determine whether the median MIC distributions in the IPD and NIPD groups were significantly different; *p* < 0.05 indicates a significant difference. *P_(K-S)_*: The Kolmogorov–Smirnov test was used to determine whether the MIC distributions in the IPD and NIPD groups were significantly different. *p* < 0.05 indicates a significant difference. Bold: This *p* value indicates that the data are significantly different.

### 3.4 Relationships between antibiotic resistance-related genes and susceptibility phenotypes

**Figure 4:**
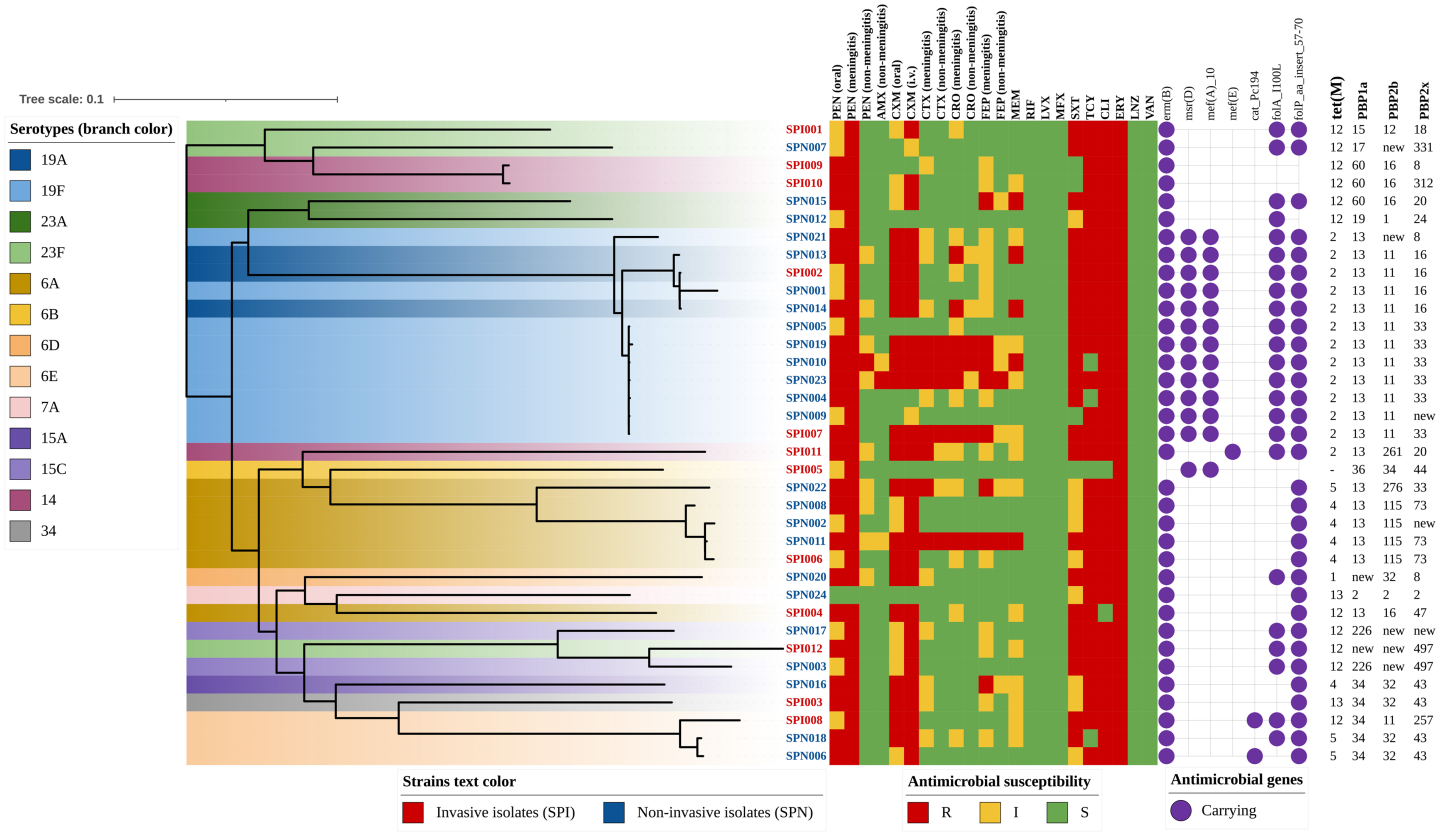
Phylogenetic tree annotated with antibiotic resistance-related genes and susceptibility phenotypes (rectangular). The color of the isolate name indicates the strain type (e.g., red text represents invasive isolates), and the branch color indicates the serotype of each strain (e.g., dark blue represents serotype 19A). The antibiotic susceptibility phenotype is indicated with colored squares, and the antibiotic resistance-related genes are indicated with purple circles. The genotypes of *tet(M)*, PBP1a, PBP2b, and PBP2x are indicated by numbers.

To visualize the relationships among the antibiotic susceptibility phenotype, antibiotic resistance-related genes and other characteristics of *S. pneumoniae* pathogenic isolates, we constructed a phylogenetic tree (rectangular) and annotated it (Figure 4). Almost all of the isolates (97.2%) carried the ribosomal protection protein-encoding gene *tet(M)*, which confers tetracycline resistance, and the MLSb phenotype gene *erm(B)*, which confers erythromycin resistance. The ABC-F subfamily protein-encoding gene *msr(D)* and the MFS-type efflux protein-encoding gene *mef(A)*, which are located on the same operon and confer macrolide resistance, were detected mainly in isolates of the serotype 19A/19F clade of *S. pneumoniae*. The resistance characteristics of trimethoprim-sulfamethoxazole were determined mainly by folA_I100L and folP_aa_insert_57–70, with carriage rates of 61.1 and 88.9%, respectively. In addition, the MFS-type efflux protein-encoding gene *mef(E)* and the plasmid-encoded *cat* gene variant *cat-Pc194* were detected in several isolates.

In terms of erythromycin resistance, a total of 4 gene profiles were found, and their associations with MICs, diseases and serotypes are shown in Table 3. In brief, when the erythromycin MIC of 16 μg/ml was used as the cutoff point, the proportions of gene profile compositions in the two isolate groups (MIC≥16 μg/ml vs. MIC<16 μg/ml) were significantly different (*p* = 0.021). Similar differences in resistance gene carriage could also be observed when the genes were grouped by serotype. The serotype 19F isolates tended to carry the resistance genes *msr(D)* and *mef(A)*, which are not frequently present in other serotypes (100.0% vs. 14.8%, *p* < 0.001), and the proportions of genes in different serotypes were also significantly different (*p* < 0.001). Moreover, no significant difference in the distribution of erythromycin resistance genes was detected between the IPD and NIPD isolates.

**Table 3:** Erythromycin resistance-associated gene carriage rates for 36 *Streptococcus pneumoniae* isolates in this study.

Cases	Total	%	Diseases					ERY MIC (μg/ml)					Serotypes				
			IPD	%	NIPD	%	*p*	MIC≥16	%	MIC<16	%	*p*	19F	%	others	%	*p*
Gene carriage																	
*erm(B)*	35	97.2	11	91.7	24	100.0	0.333	31	100.0	4	80.0	0.139	9	100.0	26	96.3	0.558
*msr(D)*	13	36.1	3	25.0	10	41.7	0.326	11	35.5	2	40.0	0.845	9	100.0	4	14.8	**<0.001**
*mef(A)*	13	36.1	3	25.0	10	41.7	0.326	11	35.5	2	40.0	0.845	9	100.0	4	14.8	**<0.001**
*mef(E)*	1	2.8	1	8.3	0	0.0	0.151	0	0.0	1	20.0	0.139	0	0.0	1	3.7	0.558
Gene profile							0.096					**0.021**					**<0.001**
*erm(B)*	22	61.1	8	66.7	14	58.3	I	20	64.5	2	40.0	I	0	0.0	22	81.5	I
*msr(D) + mef(A)*	1	2.8	1	8.3	0	0.0	I	0	0.0	1	20.0	II	0	0.0	1	3.7	I, II
*erm(B) + msr(D) + mef(A)*	12	33.3	2	16.7	10	41.7	I	11	35.5	1	20.0	I, II	9	100.0	3	11.1	II
*erm(B) + mef(E)*	1	2.8	1	8.3	0	0.0	I	0	0.0	1	20.0	II	0	0.0	1	3.7	I, II
Total	36	100.0	12	100.0	24	100.0		31	100.0	5	100.0		9	100.0	27	100.0	

“Gene carriage” shows the carriage data for each gene in the isolates (each row accounts for 100%). “Gene profile” shows the various genotype data for the isolates (the 4 rows together account for 100%). The chi-square test and Fisher’s exact test were used to evaluate whether the distributions of each gene in different diseases, ERY MICs and serotypes were significantly different. The chi-square test and Z test (using the Bonferroni method to correct the *p* value) were used to evaluate whether the distribution of each genotype in different subgroups significantly differed. Each Roman numeral represents a subset with composition ratios that were not significantly different at the *p* = 0.05 level. Bold: This *p* value indicates that the data are significantly different.

In terms of β-lactam antibiotic resistance, we analyzed the types of PBPs alleles and predicted β-lactam resistance levels on the basis of transpeptidase signatures and then compared them with the MIC method interpretation (Table 4). In brief, the consistency of the two methods in the interpretation of penicillin (meningitis and non-meningitis) was 100.0%. For second- and third-generation cephalosporins, except ceftriaxone (meningitis), the consistency was greater than 80%. However, the prediction consistency for meropenem was only 66.7%. Most strains with PBPs prediction results inconsistent with the actual phenotype were susceptible or became susceptible after increased exposure to β-lactams, despite being predicted as resistant.

**Table 4:** Relationship between the prediction of antimicrobial susceptibilities by PBPs allele type and actual susceptibilities.

**Antibiotics**	Resistant cases (rate%)		Consistency cases (rate%)	Underrated cases (rate%)	Overrated cases (rate%)
	MIC-method	PBPs-predicted			
PEN (meningitis)	35 (97.2%)	35 (97.2%)	36 (100.0%)	0 (0.0%)	0 (0.0%)
PEN (non-meningitis)	0 (0.0%)	0 (0.0%)	36 (100.0%)	0 (0.0%)	0 (0.0%)
AMX	1 (2.8%)	12 (33.3%)	25 (69.4%)	0 (0.0%)	11 (30.6%)
CXM	26 (77.8%)	32 (88.9%)	30 (83.3%)	1 (2.8%)	5 (13.9%)
CTX (meningitis)	7 (19.4%)	12 (33.3%)	27 (75.0%)	2 (5.6%)	7 (19.4%)
CTX (non-meningitis)	5 (13.9%)	8 (22.2%)	31 (86.1%)	1 (2.8%)	4 (11.1%)
CRO (meningitis)	7 (19.4%)	12 (33.3%)	29 (80.5%)	1 (2.8%)	6 (16.7%)
CRO (non-meningitis)	4 (11.1%)	8 (22.2%)	30 (83.3%)	1 (2.8%)	5 (13.9%)
MEM	5 (13.9%)	13 (63.9%)	24 (66.7%)	2 (5.6%)	10 (27.8%)

β-lactam antibiotic susceptibilities were predicted via the model of Li et al. (24) using Pathogenwatch (https://pathogen.watch). MIC method breakpoint interpretation was based on CLSI M100 (34th Edition).

### 3.5 Virulence genes

**Figure 5:**
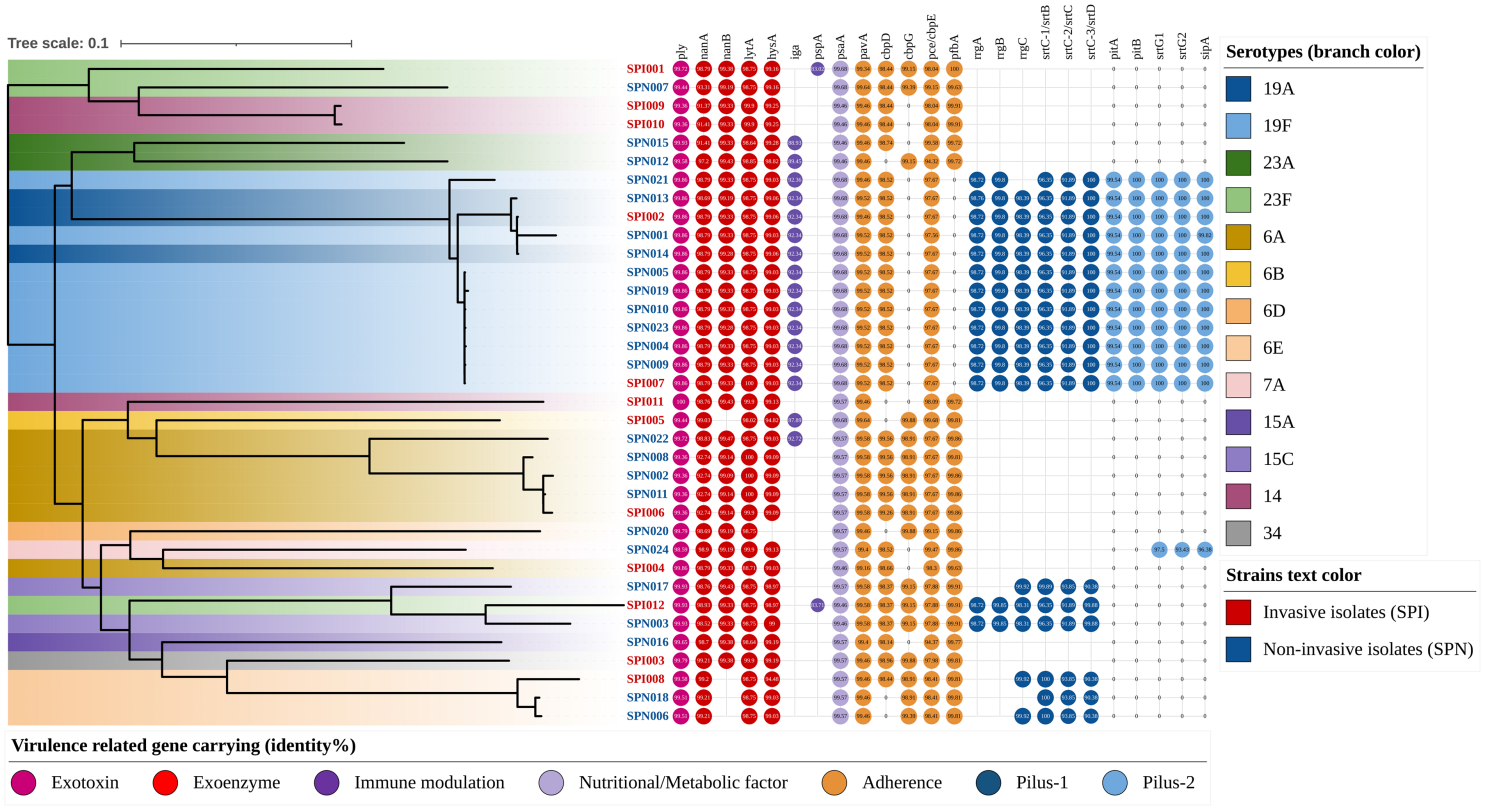
Phylogenetic tree annotated with virulence genes and serotypes (rectangular). The color of the isolate name indicates the strain type (e.g., red text represents invasive isolates), and the branch color indicates the serotype of each strain (e.g., dark blue represents serotype 19A). The virulence genes are indicated by circles, the different colors of the circles represent the different categories of virulence genes, and the central number indicates their identities compared with the reference gene sequence.

To visualize the phylogenetic characteristics among the isolate sources, serotypes and virulence genes, we constructed a phylogenetic tree (rectangular) and annotated it with gene identities (Figure 5). In total, all of the isolates carried the exotoxin gene *ply*, the exoenzyme-encoding genes *nanA* and *lytA*, the nutrition- and metabolism-related gene *psaA*, and the adherence-related genes *pavA* and *pce* (*cbpE*). Most of the isolates carried the exoenzyme-encoding genes *nanB* (88.9%) and *hysA* (97.2%) and the adherence-related gene *cbpD* (83.3%). Some of the isolates carried the immune modulation genes *iga* (44.4%) and *pspA* (5.6%), the adherence-related genes *cbpG* (47.2%) and *pfbA* (66.7%), and pilus-1 (38.9%) and pilus-2 (33.3%) related genes. The detailed information is shown in Table 5.

**Table 5:** Virulence gene carriage rates and sequence identities of 36 *Streptococcus pneumoniae* isolates in this study.

Cases	Total (*n*)	%	Seq. identity (AVG.)	Diseases								Serotypes							
				IPD (*n*)	%	Seq. identity (AVG.)	NIPD (*n*)	%	Seq. identity (AVG.)	*P_n_*	*P_Seq_*	19F (*n*)	%	Seq. identity (AVG.)	Other types (*n*)	%	Seq. identity (AVG.)	*P_n_*	*P_Seq_*
Exotoxin ^a^																			
*ply*	36	100.0	99.68%	12	100.0	99.68%	24	100.0	99.68%	-	0.993	9	100.0	99.86%	27	100.0	99.62%	-	**<0.001**
Exoenzyme ^a^																			
*nanA*	36	100.0	97.36%	12	100.0	97.17%	24	100.0	97.45%	-	0.784	9	100.0	98.79%	27	100.0	96.88%	-	**0.003**
*nanB*	32	88.9	99.33%	10	83.3	99.33%	22	91.7	99.33%	0.588	0.931	9	100.0	99.32%	23	85.2	99.33%	0.553	0.892
*lytA*	36	100.0	98.78%	12	100.0	98.45%	24	100.0	98.95%		0.747	9	100.0	98.89%	27	100.0	98.48%	-	0.743
*hysA*	35	97.2	99.06%	12	100.0	98.53%	23	95.8	99.34%	1.000	0.156	9	100.0	99.30%	26	96.3	98.98%	1.000	0.469
Immune modulation ^a^																			
*iga*	16	44.4	91.69%	3	25.0	90.86%	13	54.2	91.89%	0.157	0.296	9	100.0	92.34%	7	25.9	90.86%	**<0.001**	1.000
*pspA*	2	5.6	83.46%	2	16.7	83.46%	0	0.0	-	0.105	-	0	0.0	-	2	7.4	83.46%	1.000	-
Nutritional/Metabolic factor ^a^																			
*psaA*	36	100.0	99.59%	12	100.0	99.57%	24	100.0	99.61%	-	0.220	9	100.0	99.68%	27	100.0	99.57%	-	**<0.001**
Adherence																			
*PavA* ^b^	36	100.0	99.50%	12	100.0	99.47%	24	100.0	99.52%	-	0.111	9	100.0	99.51%	27	100.0	99.49%	-	0.341
*cbpD* ^a^	30	83.3	98.67%	10	83.3	98.61%	20	83.3	98.70%	1.000	0.548	9	100.0	98.52%	21	77.8	98.73%	0.303	0.051
*cbpG* ^a^	17	47.2	99.21%	6	50.0	99.31%	11	45.8	99.15%	1.000	0.390	0	0.0	-	17	63.0	99.21%	**0.001**	-
*pce/cbpE* ^a^	36	100.0	97.92%	12	100.0	98.28%	24	100.0	97.75%	-	0.163	9	100.0	97.66%	27	100.0	98.01%	-	0.148
*pfbA* ^b^	24	66.7	99.82%	10	83.3	99.84%	14	58.3	99.81%	0.260	0.543	0	0.0	-	24	88.9	99.82%	**<0.001**	-
Pilus-1 ^c^	14	38.9		3	25.0		11	45.8		0.292		9	100.0		5	18.5		**<0.001**	
Pilus-2 ^d^	12	33.3		2	16.7		10	41.7		0.260		9	100.0		3	11.1		**<0.001**	

This table shows the number of isolates carrying virulence-related genes (n), the proportion of virulence isolates in the subgroup (%), and the sequence identity of these virulence genes (AVG.%). a. The reference genome TIGR4 was used as a template for sequence alignment. b. The reference genome R6 was used as a template for sequence alignment. c. When rrgA, rrgB, rrgC, srtC-1, srtC-2 and srtC-3 were detected, the isolate was considered to carry pilus-1. d. When srtA, pitB, sipA, srtG1, and srtG2 were detected, the isolate was considered to carry pilus-2. *P_n_*: Chi-square tests and Fisher’s exact tests were used to analyze significant differences in the proportion of isolates with virulence genes among subgroups (with *p* = 0.05 as the cutoff level). *P_Seq_*: The t test was used to analyze significant differences in the number of base variations in the virulence genes carried by each subgroup on the basis of the reference genome (with *p* = 0.05 as the cutoff level). Bold: This *p* value indicates that the data are significantly different.

Among all the virulence-related genes detected in this study, the genes with high conservation (carriage rate > 97%) and an average identity of greater than 97% with the reference strains included the following: the exotoxin gene *ply* (carriage rate: 100.0%, identity: 99.68%), the exoenzyme-encoding genes *nanA* (carriage rate: 100.0%, identity: 97.36%), *lytA* (carriage rate: 100.0%, identity: 98.78%) and *hysA* (carriage rate: 97.2%, identity: 99.06%), the nutrition- and metabolism-related gene *psaA* (carriage rate: 100.0%, identity: 99.59%), and the adherence-related genes *pavA* (carriage rate: 100.0%, identity: 99.50%) and *pce/cbpE* (carriage rate: 100.0%, identity: 97.92%). Detailed information on the gene carriage rates and sequence identities is shown in Figure 5 and Table 5.

The gene carriage rates and sequence identities of some virulence-related genes of 19F serotype isolates were significantly different from those of other serotypes. The genes that were significantly more common in the serotype 19F isolates than in the other isolates included the following: the immune modulation gene *iga* (100.0% vs. 25.9%, *p* < 0.001), pilus-1-related genes (100.0% vs. 18.5%, *p* < 0.001) and pilus-2-related genes (100.0% vs. 11.1%, *p* < 0.001). However, the adherence-related gene *pfbA* was not detected in serotype 19F isolates (0.0% vs. 88.9%, *p* < 0.001). Among the virulence-related genes detected in this study, the genes with significantly greater sequence identity in serotype 19F isolates than in other serotypes included the exotoxin gene *ply* (99.86% vs. 99.62%, *p* < 0.001), the exoenzyme-encoding gene *nanA* (98.79% vs. 96.88%, *p* = 0.003), and the immune modulation gene *pspA* (99.68% vs. 99.57%, *p* < 0.001). The detailed evaluation and information are shown in Table 5 and its footnote.

## 4 Discussion

In this study, we not only evaluated the MICs of 16 antimicrobial agents and interpreted the sensitivity breakpoints on the basis of 22 rules but also conducted whole-genome next-generation sequencing (NGS) to comprehensively analyze the molecular characteristics, including serotypes, STs, resistance-related gene carriage and variation, and virulence-related gene carriage and variation, of each pathogenic isolate in Southwest China during the second outbreak of COVID-19 that occurred in this area. Prior to this period, the government had implemented a strict “zero infection” policy for COVID-19 for several years while also eliminating the transmission routes of various other respiratory pathogens. The isolation rate of *S. pneumoniae*, one of the most common respiratory pathogens in children, decreased sharply during the control period. However, when this strict policy ended, the number of cases of pneumococcal infection, including invasive infections, in children began to increase again. Therefore, after 3 years of pandemic prevention and control, the development of antibiotic susceptibility and molecular characterization of pathogenic *S. pneumoniae* isolates has become a topic of interest and concern.

The prevalent serotypes in IPD patients were 14, 6A, and 23F, and the prevalent serotypes in NIPD patients were 19F and 6A. A significant difference in the proportion of serotype 14 strains is noted in the two groups, which was similar to the data obtained before the outbreak of COVID-19 from 2017 to 2019 (10). In the early 2020s, the prevalent serotypes of pneumococcal disease were 19F (27.2%), 6A/B (19.1%), 23F (11.0%), and 14 (8.4%) in East China (26), and the prevalent serotypes were 19F (23.4%), 23A (8.9%), 23F (7.8%), and 6B (7.8%) in Southeast China (27). Although the main prevalent serotypes of pneumococcal pathogenic isolates in China, an East Asian country with a population of 1.4 billion, were still 19A/F, 6A/B and 23A/F during this period, the proportions of subsequent specific serotypes were changing. For *S. pneumoniae* isolated from pediatric patients, the primary selective pressure faced by different serotypes is immunization via pneumococcal conjugate vaccines (PCVs). PCVs are self-purchased vaccines for residents in mainland China and are not included in the national medical benefits. Therefore, the PCV vaccination rate is related to local economic development and residents’ income level. Currently, the only legal PCV in mainland China is the 13-valent pneumococcal conjugate vaccine (PCV13). In Shenzhen, which is one of the four most economically developed cities in mainland China and the city closest to Hong Kong, approximately 46.6% of the *S. pneumoniae* isolates were not covered by serotypes of the PCV-13 vaccine (nonvaccine serotypes, NVTs) (27), and this value is higher than the values reported in other areas of mainland China. A similar “serotype replacement” phenomenon caused by vaccination is also occurring in Taiwan. Since the Taiwan authorities started vaccinating all eligible children with PCV-13 in 2015, the most prevalent serotypes of *S. pneumoniae* have shifted to 15A and 23A, both of which are NVTs (28). In the United States, which has a long history of PCV-13 vaccination, there has also been a significant increase in the isolation rate of NVTs 23B and 15A (29), which has also been observed in the United Kingdom, with the most prevalent serotypes being 15 B/C and 11A (30). After the COVID-19 pandemic, NVTs 24F and 8 ranked among the top three serotypes responsible for invasive infections in children in Spain (6, 31) and Portugal (32), respectively. On the one hand, the promotion and application of PCVs has indeed reduced the incidence of pneumococcal diseases in children, especially invasive pneumococcal infections resistant to first-line antibiotic treatments (5). On the other hand, long-term prevention and immunization against specific serotypes has increased the morbidity of other serotypes, forcing the development of PCVs covering more serotypes (6). PCV-20 will soon be launched in China, and PCV-24 has also entered the phase III clinical trial stage and is expected to curb the replacement of serotypes caused by PCV-13 to a certain extent. However, more than one hundred serotypes of *S. pneumoniae* have been discovered. The upper limit of the number of antigens and the corresponding cost of vaccine programs designed for polysaccharide capsules with different configurations are problems that must be resolved via further vaccine research and development. Perhaps in the next decade, there will be a breakthrough in research on and the development of protein vaccines or nucleic acid vaccines targeting conserved proteins or other antigenic sites of *S. pneumoniae*.

After the COVID-19 pandemic, CC320 (GPSC 1) remained the most prevalent CC in this area, and novel STs accounted for 11.1% of all isolates (4/36), which is higher than the number reported for children from 2017 to 2019 (6.25%) (10) but lower than the number reported for the overall population from 2018 to 2022 (35.51%) (9). These findings indicate that after 3 years of strict isolation and treatment of pneumonia patients, the genomic evolution of *S. pneumoniae* has not stagnated, and it still maintains a certain mutation rate. The upper respiratory tract of the healthy population is considered the main source of infection and bacterial reservoir of *S. pneumoniae* (1) and may also serve as the main site for the genetic recombination of *S. pneumoniae*. Simple population control measures that focus on controlling patients and cutting off transmission routes can reduce the infection rate of *S. pneumoniae* during the policy implementation period, but they are unlikely to prevent *S. pneumoniae* from evolving in the population and quickly returning to its original level after the isolation policy ends. Thus, measures to protect susceptible populations, mainly vaccination-based methods, appear to be more effective in preventing pneumococcal disease.

Given that the penetration ability of pneumococcal vaccines is currently insufficient, the rational use of antibiotics has become the primary measure for preventing and treating pneumococcal infection. For a long time, penicillins and cephalosporins have been the first-line antibiotics for the empirical treatment of *S. pneumoniae* and other transmitted bacterial respiratory pathogens in mainland China. In this study, for non-meningitis patients, regardless of whether they had invasive infections, the resistance proportions remained low, even though some pathogenic isolates were nonsusceptible to penicillin and amoxicillin. The nonsusceptibility rate to penicillins was higher than that reported in recent research in Australia (PEN: 2.6%, AMX: 2.6%) (33) and Canada (PEN: 1.4%) (34) but lower than that reported in Taiwan (PEN: 43.41%) (28) and much lower than that reported in Vietnam (PEN: 100.0%) (35).

Variation in penicillin-binding proteins (PBPs) is the leading cause of the resistance of *S. pneumoniae* isolates to penicillins, cephalosporins, and carbapenems. When the structures of PBPs change, these *β*-lactam antibiotics cannot effectively bind to them and thus cannot inhibit the formation of peptidoglycan in the cell wall, rendering bactericidal activity ineffective (36). Li et al. developed a classification system in which a pneumococcal isolate was assigned to a “PBP type” on the basis of sequence signatures in the transpeptidase domains (TPDs) of the three critical PBPs (PBP1a, PBP2b, and PBP2x) and further predicted the susceptibility of the isolates to β-lactams (24). In this study, we used the Pathogenwatch website to invoke the model for prediction, and the interpretation of penicillin (meningitis and non-meningitis) were 100% consistent with the traditional MIC identification method. However, for other β-lactam antibiotics, the prediction consistency of the PBPs method decreased, and the false positive rate for resistant strains increased. The possible reason for the decline in prediction consistency is that a considerable number of new PBPs-type strains (8/36, 22.2%) were found among the strains isolated in this study, making it difficult for the previously established prediction model to obtain accurate interpretations.

For patients with meningitis, we should consider not only the sensitivity of pathogenic isolates to antibiotics but also the ability of antibiotics to penetrate the blood–brain barrier and whether sufficient drug concentrations can be achieved in the brain. The CLSI penicillin resistance MIC breakpoint for patients with meningitis is set at 0.06 μg/ml, which is much lower than the MIC breakpoint of 8 μg/ml for patients without meningitis. In this study, according to the interpretation criteria for meningitis, almost all the isolates were penicillin resistant, and only approximately half of the isolates were susceptible to cefotaxime, ceftriaxone, and cefepime. Considering that *S. pneumoniae* is common in China and that the isolates are highly resistant to penicillin and third-generation cephalosporins, the Chinese guidelines recommend the use of third-generation cephalosporins plus vancomycin as the initial empirical treatment for bacterial meningitis (37).

Macrolide antibiotics, including erythromycin, were once widely abused in China given their broad antibacterial spectrum and ability to kill atypical pathogens, including Mycoplasma and Chlamydia, which has led to widespread resistance of *S. pneumoniae* to erythromycin. In this study, all of the isolates were resistant to erythromycin, which was similar to previous reports in China (9, 38, 39). The resistance of *S. pneumoniae* to macrolides occurs mainly through the targeted modification of ribosomes by the ribosomal methyltransferase encoded by *erm(B)*, thereby reducing the binding of macrolides. The expression of *erm(B)* can be induced by erythromycin. The leader peptide, which causes mRNA attenuation and stabilizes its structure to prevent further translation, undergoes a change in conformation in the presence of erythromycin, allowing the expression of *erm(B)*. In addition, *mef(A/E)* encodes MFS-type efflux proteins, which are located on the same operon as *msr(D),* which encodes an ABC-F subfamily protein. Together, they enhance the elimination of macrolides and increase resistance. In this study, almost all the isolates carried *erm(B)*, and more than one-third of the isolates carried *msr(D)* and *mef(A/E)*, indicating that both resistance mechanisms are the main causes of the high macrolide resistance of *S. pneumoniae* in China. Notably, after the second wave of the COVID-19 pandemic in China, from late 2023 to early 2024, *Mycoplasma pneumoniae* pneumonia was widely prevalent in pediatric patients, and the prevalent Mycoplasma strains were generally resistant to macrolides, forcing the use of quinolones, which exhibit joint and cartilage toxicity, for treatment (40).

The polysaccharide capsule and protein virulence factors are the two main sources of virulence in *S. pneumoniae*. In the virulence gene analysis, all the isolates carried capsule-related genes, and their serotypes were successfully identified. Among the other virulence genes, *ply* encodes pneumolysin, which is the major exotoxin of *S. pneumoniae*. As a cholesterol-dependent cytolysin (CDC), pneumolysin acts on cholesterol-rich biofilms, disrupts tight junctions between endothelial cells, inhibits the cilia of the bronchial epithelium, destroys the mechanical barrier of the respiratory tract, promotes the adhesion of isolates to the bronchial epithelium and accelerates infection. Moreover, pneumolysin can damage the epithelial structure of alveolar capillaries, promote isolate entry into the blood, and cause invasive pneumococcal diseases (41). In this study, *ply* also exhibited sequence conservation, with sequence identity among strains >99%. The pneumolysin derivative PlyD1 is safe and immunogenic and is an important candidate protein vaccine that is expected to overcome the serotype limitations of existing pneumococcal polysaccharide conjugate vaccines (42, 43).

Among the other highly conserved virulence factors, neuraminidase A, which is encoded by the *NanA* gene, cleaves terminal sialic acid residues from oligosaccharide receptors in host epithelial cells to facilitate colonization by *S. pneumoniae* (44). The lytic amidase LytA, which is encoded by the *lytA* gene, is a peptidoglycan hydrolase anchored on the bacterial cell wall that can hydrolyze the amide bond between N-acetylmuramic acid and L-alanine and plays a key role in bacterial autolysis (45). Hyaluronidase A, which is encoded by the *hysA* gene, is an LPXTG-anchored surface protein that facilitates tissue invasion by breaking down extracellular matrix (ECM) components (46). Pneumococcal surface antigen A, which is encoded by the *PsaA* gene, anchors to the bacterial membrane via a lipid attached to the protein and transports Mn^2+^ and Zn^2+^ into the cytoplasm (47). Pneumococcal adherence and virulence factor A, which is encoded by the *pavA* gene, acts directly as a fibronectin adhesin and affects pneumococcal colonization by modulating the expression or function of virulence determinants (48). The choline binding protein E, which is encoded by the *pce/cbpE* gene, is involved in the distribution and restructuring of the content of choline in the cell wall and indirectly modulates the activity of other CBPs and pathogen–host interactions (49). Given that these virulence factors are widely distributed and highly conserved in *S. pneumoniae*, they have also attracted attention in the development of vaccines and diagnostic reagents. In recent years, studies have reported that these compounds and their derivatives have strong immunogenicity and vaccine potential, can be further developed as combined antigen vaccines or fusion protein vaccines (50–53), and can be used as PCR detection targets in the diagnosis of pneumococcal disease (54, 55).

In this study, no significant difference was detected between the virulence gene carriage rates of *S. pneumoniae* isolates causing invasive infection and noninvasive infection, nor was there any sequence variation. However, for serotype 19F isolates, their *iga* carriage rate was significantly greater than that of other serotype isolates, which means that they were more likely to express IgA1 protease, which cleaves the Pro227-Thr228 peptide bond in the IgA1 hinge and may enable *S. pneumoniae* to subvert the antigen specificity of the humoral immune response to facilitate adhesive interactions and persistence on the mucosal surface (56). Moreover, almost all serotype 19F isolates carried both types of pili, which gives them an advantage in competing with other serotype isolates for adhesion and invasion and makes it easier for them to overcome host defenses and participate in biofilm formation (57). Therefore, this result indirectly confirms that the promotion of PCV-13 in Southwest China may be beneficial, as serotype 19F is a vaccine serotype (VT).

Our study focused on invasive infections with *S. pneumoniae* in pediatric patients in Southwest China from August 2022 to September 2023, when the second outbreak of COVID-19, dominated by the Omicron variant, occurred in this area. During this period, almost all medical resources were focused on the prevention, control and treatment of COVID-19. In this context, we continued to culture, purify, preserve, sequence and analyze clinically invasive and noninvasive *S. pneumoniae* isolates, revealing their antibiotic susceptibility and performing molecular characterization during this unique historical period. However, the primary limitation of this study is that the sample size for invasive strains was small, and the results of the analysis were mostly limited to descriptive results. Considering the timeliness of the data and despite the shortcomings of the study, the findings still provide a theoretical basis for the prevention and treatment of *S. pneumoniae* in this new-normal period with respect to COVID-19, and we will continue to pay attention to its epidemic status in this area.

In conclusion, this study provides valuable information on the antibiotic susceptibility and molecular prevalence of pediatric invasive and noninvasive *S. pneumoniae* isolates during the second outbreak of COVID-19 in Southwest China. The most prevalent serotypes in IPD patients were 14, 23F and 6A, and the prevalent CCs were CC320 (GPSC 1), CC199 (GPSC 4), and CC902 (GPSC 321). Moreover, 19F remained the most prevalent serotype in respiratory isolates. Considering the prevalence of antimicrobial resistance, a third-generation cephalosporin combined with vancomycin can be used to treat pediatric patients with invasive infections, especially meningitis. Alternatively, levofloxacin or moxifloxacin can be used instead if necessary. Benefits can be expected from vaccinating infants with pneumococcal conjugate vaccines, and conserved virulence factors may also contribute to the development of next-generation vaccines.

## Data Availability

The datasets presented in this study can be found in online repositories. The names of the repository/repositories and accession number(s) can be found at: https://www.ncbi.nlm.nih.gov/genbank/, PRJNA1045333.

## Footnotes

^1^ https://pathogen.watch/

^3^ https://github.com/BenJamesMetcalf/Spn_Scripts_Reference

^4^PubMLST. (2024). https://pubmlst.org/organisms/streptococcus-pneumoniae.
